# Case report: Endolymphatic sac tumor with blurred vision

**DOI:** 10.3389/fonc.2024.1463526

**Published:** 2025-01-13

**Authors:** Ke Yuan, Weijian Luo, Jia Chen, Quanzhou Peng, Xiaoguang Tong, Jiliang Hu

**Affiliations:** ^1^ The Second Clinical Medical College of Jinan University, Shenzhen, China; ^2^ The First Affiliated Hospital of the Southern University of Science and Technology, Shenzhen, China; ^3^ The Department of Neurosurgery, Shenzhen People’s Hospital, Shenzhen, China; ^4^ Guangdong Engineering Technological Research Center for Nervous Anatomy and Related Clinical Applications, Shenzhen, China; ^5^ The Department of Pathology, Shenzhen People’s Hospital, Shenzhen, China; ^6^ Department of Neurosurgery, Tianjin Huanhu Hospital, Tianjin, China; ^7^ Department of Neurosurgery, Tianjin Central Hospital for Neurosurgery and Neurology, Tianjin, China; ^8^ Clinical College of Neurology, Neurosurgery and Neurorehabilitation, Tianjin Medical University, Tianjin, China; ^9^ Laboratory of Microneurosurgery, Tianjin Neurosurgical Institute, Tianjin, China; ^10^ Tianjin Key Laboratory of Cerebral Vascular and Neural Degenerative Diseases, Tianjin, China

**Keywords:** endolymphatic sac tumor, microsurgery, adjuvant radiotherapy, cerebellopontine angle tumor, Von Hippel-Lindau disease (VHL)

## Abstract

**Introduction:**

Endolymphatic sac tumor (ELST) is a rare neoplasm that exhibits aggressive growth primarily in the endolymphatic capsule and can potentially affect nearby neurovascular structures. The diagnosis of ELST poses challenges due to its low prevalence, gradual progression, and nonspecific symptomatology. It is currently believed that prompt surgical intervention is recommended for endolymphatic sac tumors upon diagnosis. In cases where the lesion is complex or in close proximity to vital blood vessels and nerves, making complete resection challenging, adjuvant therapy may be employed postoperatively. This approach aims to enhance treatment outcomes.

**Case presentation:**

A case of a 53-year-old male was admitted to the Department of Neurosurgery of Shenzhen People’s Hospital with the main cause of dizziness and blurred vision, and was found to have an occupation in the pontocerebellar angle for about 20 days. Preoperative cranial CT suggested that the temporal bone mastoid was widely damaged with worm-like bone, and some of them showed honeycomb changes. MRI suggested that shadow was seen in the posterior part of the left internal auditory canal, temporal bone mastoid and jugular foramen, with a size of about 28x21x27mm.The T1-weighted image showed low and equal signals, and the T2-weighted image showed equal and slightly low signals in the center with multicompartmental cystic high signals in the margin. The center of the mass strengthened significantly after enhancement. The patient had no other clinical manifestations and no family history. The clinical diagnosis was left-sided pontocerebellar angle occupation - nature to be determined. The occupation was resected microscopically using the distal lateral combined anterior approach to the sigmoid sinus. Due to the extremely rich blood supply of the tumor, the tumor was embedded in the petrous humerus, which was soft and tough, and the surrounding structures were not clearly displayed, making surgical resection extremely difficult. postoperative pathology and immunohistochemistry confirmed that this lesion was an endolymphatic cystic tumor. After surgery, most of the tumor was successfully resected while preserving neurological function well, and the residual tumor was treated with adjuvant gamma knife with good results.

**Conclusion:**

ELST is a rare, low-grade, locally aggressive tumor that is difficult to diagnose early. During the surgery, it was observed that the tumor had a significant blood supply, which made its removal challenging. Preoperative embolization of the tumor’s blood supply artery would facilitate surgical resection and help avoid massive intraoperative bleeding. Complete surgical resection is the treatment of choice, and any remaining tumor remnants can be managed with adjuvant radiotherapy post-surgery, necessitating long-term follow-up to monitor any developments.

## Introduction

The endolymphatic sac is derived from the neural ectoderm and is located posteromedial region of the temporal bone, positioned between the internal auditory canal and the ethmoid sinus (1). The labyrinth plays a crucial role in regulating the volume and pressure of endolymph, participating in the immune response of the inner ear, eliminating waste products from the endolymph through phagocytosis (2, 3), and maintaining vestibular function. Endolymphatic sac tumor (ELST) is a rare tumor that invades the inner ear. Most ELSTs occur sporadically, but some are associated with VHL disease, an autosomal dominantly inherited condition. ELSTs are characterized by slow growth and are often asymptomatic in the early stages. Imaging studies typically do not show any notable features. When there is evident peripheral tissue destruction, distinguishing it from associated lesions in the neighboring organs becomes exceedingly challenging. The resection of the tumor during surgery poses significant challenges due to several factors. These include the tumor’s abundant blood supply, its variable softness and toughness, and the limited visibility of the surrounding structures. Our team encountered a challenging case with a difficult preoperative diagnosis. During the intraoperative resection of the tumor, significant bleeding was observed and the tumor structure was unclear. However, postoperative resection successfully removed most of the tumor, and the remaining tumor was treated with gamma knife assistance. The results of the treatment were favorable, and are reported as follows.

## Case presentation

**Figure 1:**
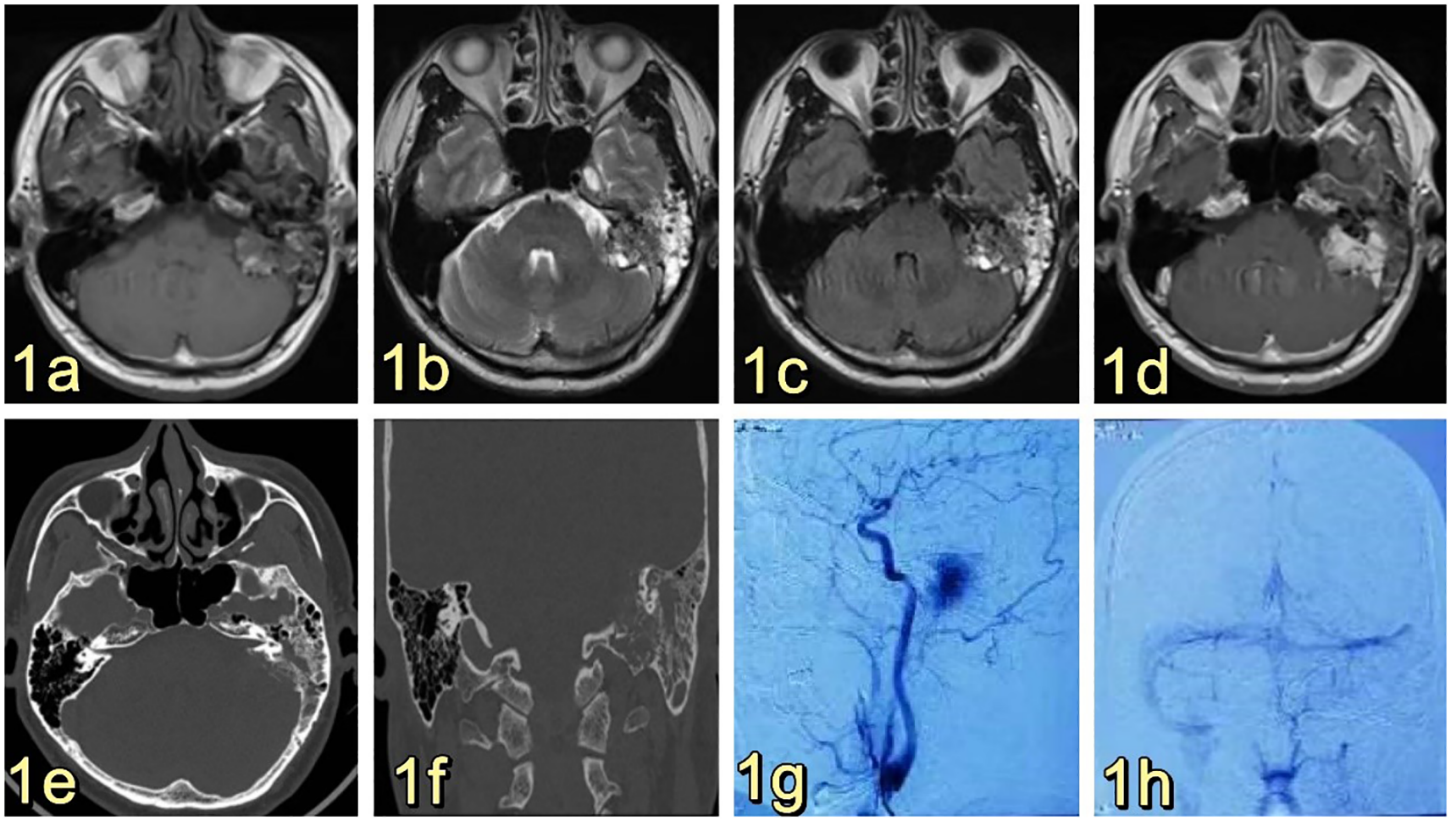
**(A)** Presents a cranial MRI image of the left side of the posterior internal auditory canal and temporal bone mastoid region, showing an irregular mixed signal with dimensions of approximately 28x21x27mm. The T1W1 signal is low and equal, and a high signal ring can be observed at the edge of the lesion, with a peripheral morphology that is not well-defined and foliated. **(B, C)** display T2W1 and FLAIR images, respectively, revealing an equal and slightly low signal at the center, along with multiple cystic high signals at the edge. **(D)** demonstrates obvious enhancement of the mass in the center, accompanied by an increase in signal in the left mastoid region, and the presence of a flowing void effect can be observed. **(E)** and **(F)** are not provided. After undergoing enhancement, there was a significant strengthening of the center of the mass. Additionally, there was an increase in the signal of the left mastoid, and the presence of a vascular void signal could be observed **(E, F)**. Ear/mastoid/temporal bone CT scan revealed extensive worm-eaten bone destruction in the mastoid region of the temporal bone **(G, H)**. Transverse common carotid artery angiography demonstrated abnormal visualization of the left posterior cranial fossa, with the left pharyngeal ascending artery supplying blood to the tumor and exhibiting a rich blood supply **(G)**. Furthermore, left vertebral angiography indicated that there was no visualization of the left transverse sinus and the ethmoid sinus **(H)**. The left vertebral imaging revealed a lack of visualization of the left transverse sinus and sigmoid sinus after one hour.

**Figure 2:**
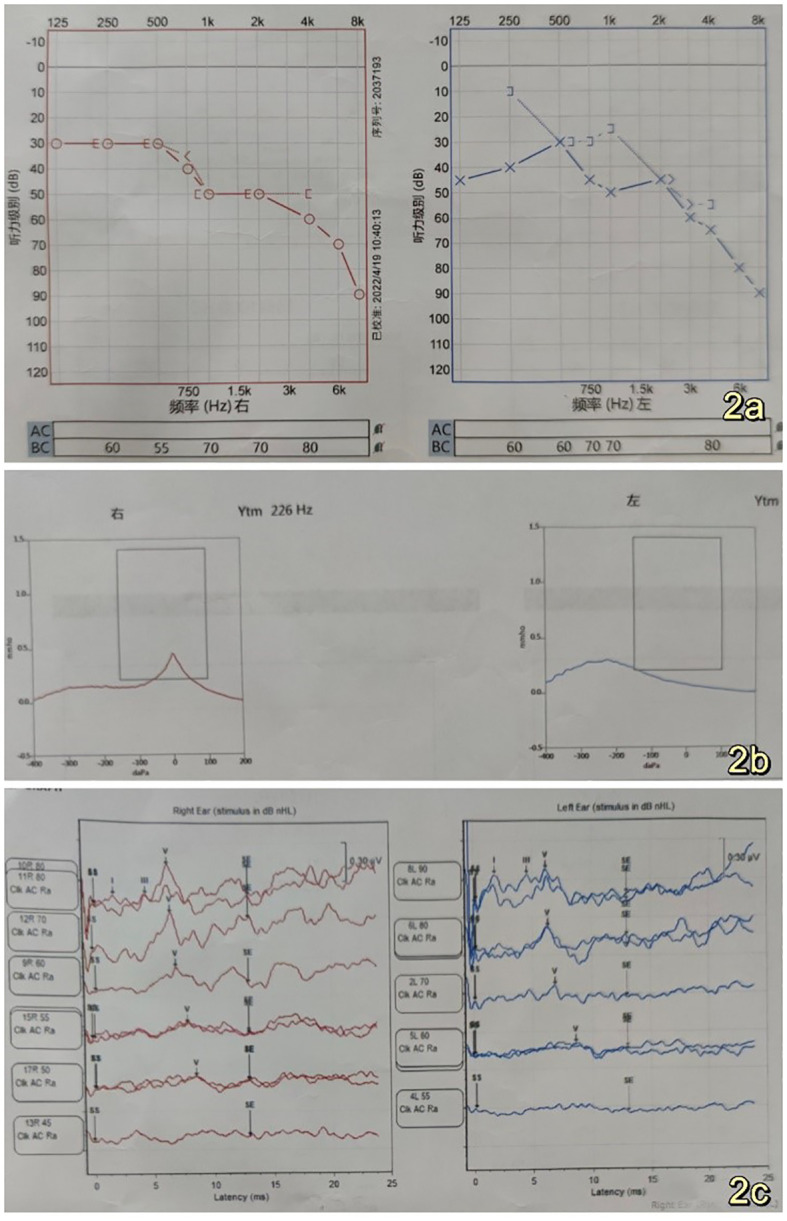
**(A)** Left and right ear air-conducted short acoustic auditory brainstem response (ABR) thresholds were measured at 60 dBnHL and 50 dBnHL, respectively, indicating the presence of moderate sensorineural hearing loss in both the left and right ears **(B)**. Acoustic conductance exhibits a C-mode pattern in the left ear and an A-mode pattern in the right ear **(C)**. Stapedial reflexes were not observed in the left ear, while a stapedial reflex was observed in the right ear **(C)**. The left ear did not respond, while the right ear did respond.

The 53-year-old male patient was admitted to the Department of Neurosurgery at Shenzhen People’s Hospital on February 19, 2023, presenting with symptoms of dizziness and blurred vision. Further examination revealed the presence of a pontine cerebellar angle occupation, which had been present for approximately 20 days. The patient was admitted to a foreign hospital 20 days ago due to symptoms of dizziness and blurred vision. A craniocerebral CT scan revealed the presence of a neoplastic lesion in the left pontocerebellar angle, accompanied by osteoporosis of the left temporal bone. Cranial MRI scanning with enhancement reveals the presence of a lesion in the left pontocerebellar angle, specifically involving the left mastoid cone. This finding raises the possibility of an extracerebral tumor, with a potential diagnosis of mesenchymal meningioma. The patient reported no previous instances of dizziness and headache, tinnitus, unsteady walking, surgery, or trauma. Additionally, there was no family history of Von Hippel-Lindau disease (VHL). Physical examination revealed patent external auditory canals bilaterally, bilateral coarse hearing loss, and no abnormalities in cerebral neurologic function. Preoperative cranial magnetic resonance imaging (MRI) revealed an irregular mixed-signal located in the posterior region of the left internal auditory canal, the mastoid process of the temporal bone, and the jugular foramen. The size of the mass was approximately 28x21x27 mm. T1W1 imaging demonstrated uniform and diminished signals, while the periphery of the lesion exhibited a high signal in the form of a ring, displaying an irregular lobular morphology (**Figure 1A**). T2-weighted imaging (T2W1) (**Figure 1B**) and fluid-attenuated inversion recovery (FLAIR) (**Figure 1C**) demonstrated a central region with uniform and slightly decreased signal intensity, while the periphery of the lesion exhibited a multicompartmental cystic high signal. The center of mass exhibited a noticeable increase in strength following the implementation of enhancements. After enhancement, there was a clear strengthening of the center of mass. Additionally, there was a slight compression of the adjacent left sigmoid sinus, partial encirclement of the left internal jugular vein by the mass, thinning of the internal jugular vein, and an increase in signal intensity in the left mastoid. Vascular flow void signal was observed in all images, as depicted in **Figure 1D**. CT imaging, along with 3D reconstruction, revealed evidence of bony structure damage in the left mastoid and a portion of the posterior semicircular canal. However, the visualization of the stapes was not captured in the imaging. The mastoid airspace exhibited partial fusion and enlargement, accompanied by an increase in density within the region. The mastoid bone of the temporal bone exhibits extensive damage characterized by worm-like bone destruction and honeycomb changes. The external auditory canal remains open, while the internal auditory canal shows slight dilation. The surrounding brain tissue exhibited a slight decrease in density, as observed in **Figures 1E, F**. In our department, a digital subtraction angiography (DSA) procedure was conducted, revealing abnormal visualization of the left posterior cranial fossa through the left common carotid artery. Additionally, the left ascending pharyngeal artery was found to supply blood to the tumor and exhibited a significant blood supply (**Figure 1G**). Furthermore, imaging of the left vertebral artery indicated a lack of visualization of the left transverse sinus and sigmoid sinus (**Figure 1H**). Pure tone audiometry and acoustic conductance impedance were conducted to assess the auditory brainstem response (ABR) thresholds for air-conducted short sounds in both the left and right ears. The results indicated that the ABR response threshold for the left ear was 60 dbnHL, while for the right ear it was 50 dbnHL. These findings suggest the presence of moderate sensorineural deafness in both the left and right ears. (**Figure 2A**) displays the acoustic conductance impedance, revealing a C-mode map in the left ear and an A-mode map in the right ear (**Figure 2B**). Additionally, the stapedius muscle reflex was not elicited in the left ear, while it was elicited in the right ear (**Figure 2C**).

**Figure 3:**
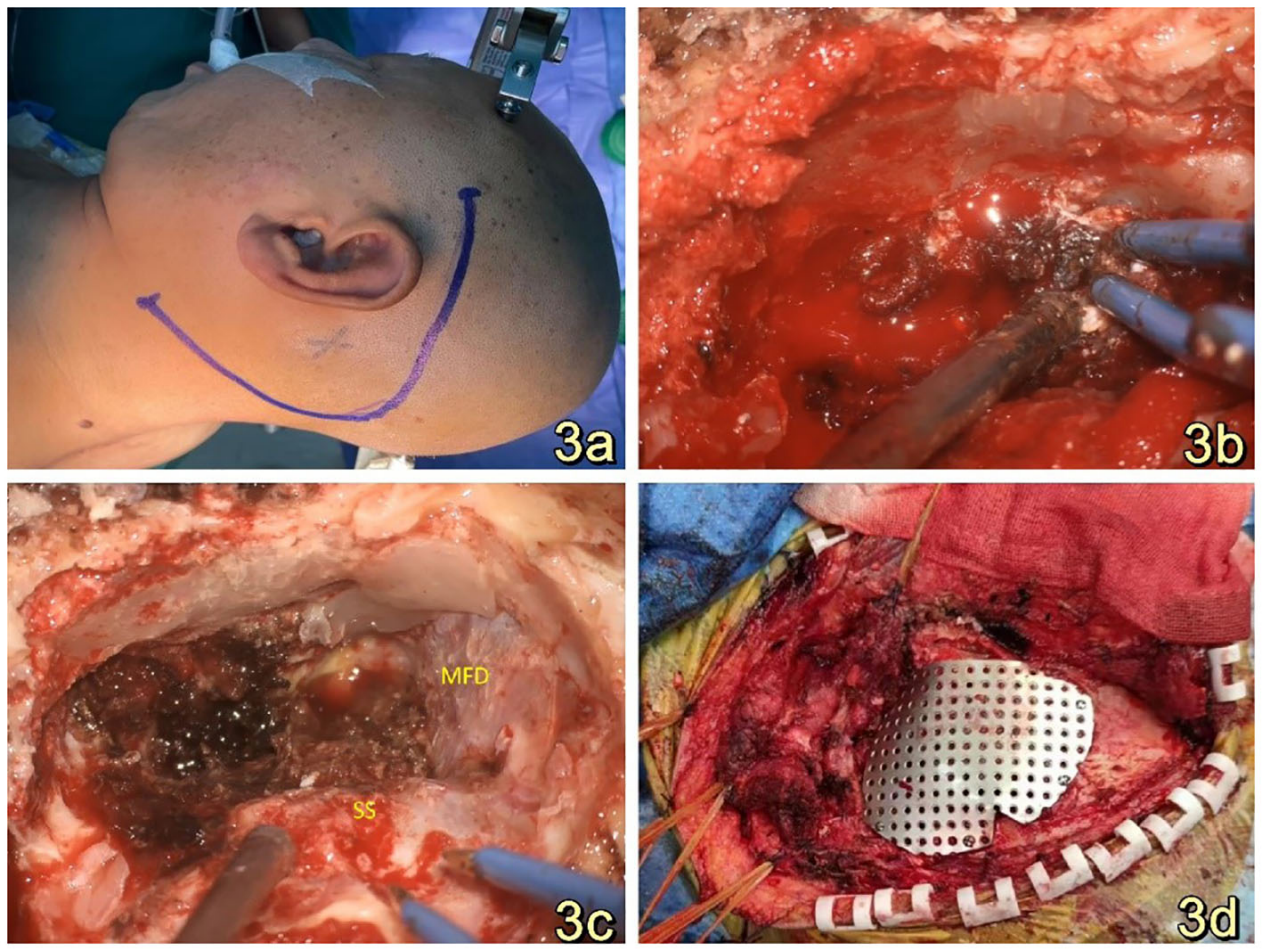
**(A)**: A substantial C-shaped incision was performed posterior to the left ear while the patient was positioned in the right lateral recumbent position. **(B)**: The tumor exhibited a grayish-red hue, possessed a firm texture, and displayed a significant vascularization, necessitating intraoperative hemostasis with the use of gelatin sponges. **(C)**: A panoramic view of the resected tumor reveals that the temporal floor dura and sigmoid sinus remained intact. The tumor cavity was situated in the deeper section of the rocky bone aponeurosis. This three-dimensional representation provides a comprehensive visualization of the surgical outcome. **(D)**: Titanium plates were utilized for the purpose of repairing the bony defects of the mastoid processes, as well as serving as attachment points for the repositioning of the retrooccipital musculature.

**Figure 4:**
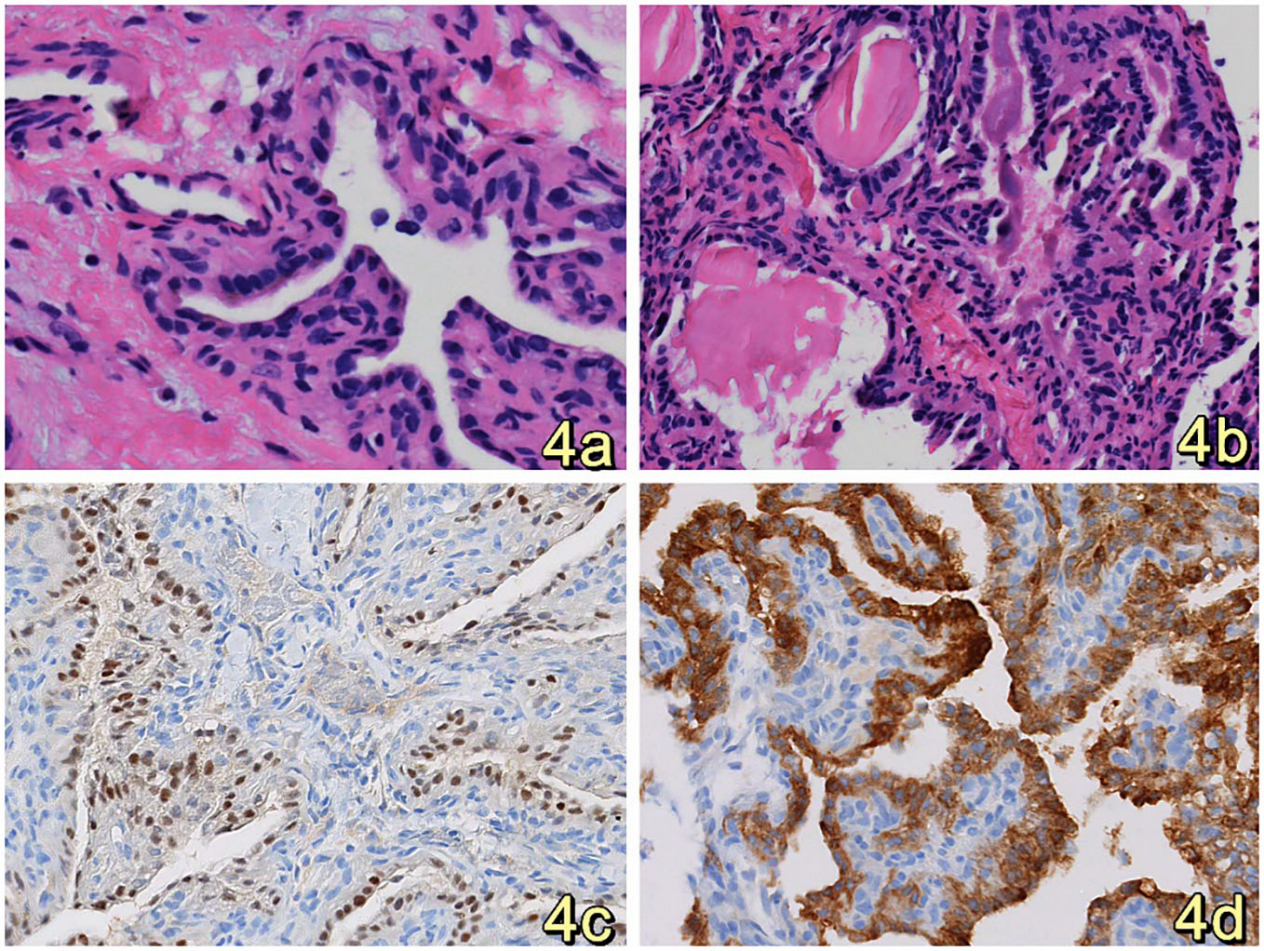
**(A, B)**. HE staining revealed that the tumor cells were organized in a papillary, glandular pattern within an enlarged lumen. The cells had round nuclei and abundant, eosinophilic, granular cytoplasm. (Original magnification: 400×) **(C)** Immunohistochemistry analysis revealed that tumor cells diffusely expressed SOX10.(Original magnification: 400×) **(D)** Immunohistochemistry showed diffuse expression of EMA by tumor cells, suggesting consideration of inner ear lymphocystoma. (Original magnification: 400×).

Clinical diagnosis of a space-occupying lesion in the left pontocerebellar region, nature to be determined. The procedure involved a large C-shaped incision made behind the left ear (**Figure 3A**), along with a distal lateral combined anterior approach to the sigmoid sinus. During the surgical exploration, it was observed that the tumor cavity was situated in the deep region of the petrous humerus. The tumor exhibited a highly abundant blood supply, characterized by surface hemorrhage, a grayish-red color, and a firm texture (**Figure 3B**). After sufficient exposure and total excision of the tumor, the temporal dura and sigmoid sinus were adequately visualized (**Figure 3C**), and the tumor cavity was packed with periumbilical fat to prevent any potential cerebrospinal fluid leakage. The utilization of a titanium plate was employed to address the mastoid bone defect, simultaneously serving as a reattachment site for the posterior occipital muscle group (**Figure 3D**). The specimen, measuring 1.5cm*1.2cm*0.5cm, The report showed epithelial tumors consistent with endolymphatic cystic tumors (**Figures 4A–D**). The tumor cells exhibited a papillary and glandular arrangement, characterized by rounded nuclei, abundant cytoplasm, and eosinophilic and granular features (Figs. The observed characteristics of the cells include mild anisotropy, as well as rare instances of nuclear schizophrenic images (**Figures 4A, B**). Immunohistochemical analysis revealed a diffuse positive reaction for epithelial membrane antigen EMA (**Figure 4C**), CK7, CK8-18, PAX-8, S0X10 (**Figure 4D**), PAX-2. Additionally, some cells exhibited positive staining for P53, Ki-67, CD117, and S-100, as well as PR, Syn, CgA, TTF-1, P63, Tg, CK20, and CDX2. However, SATB2 immunoreactivity was negative.

**Figure 5:**
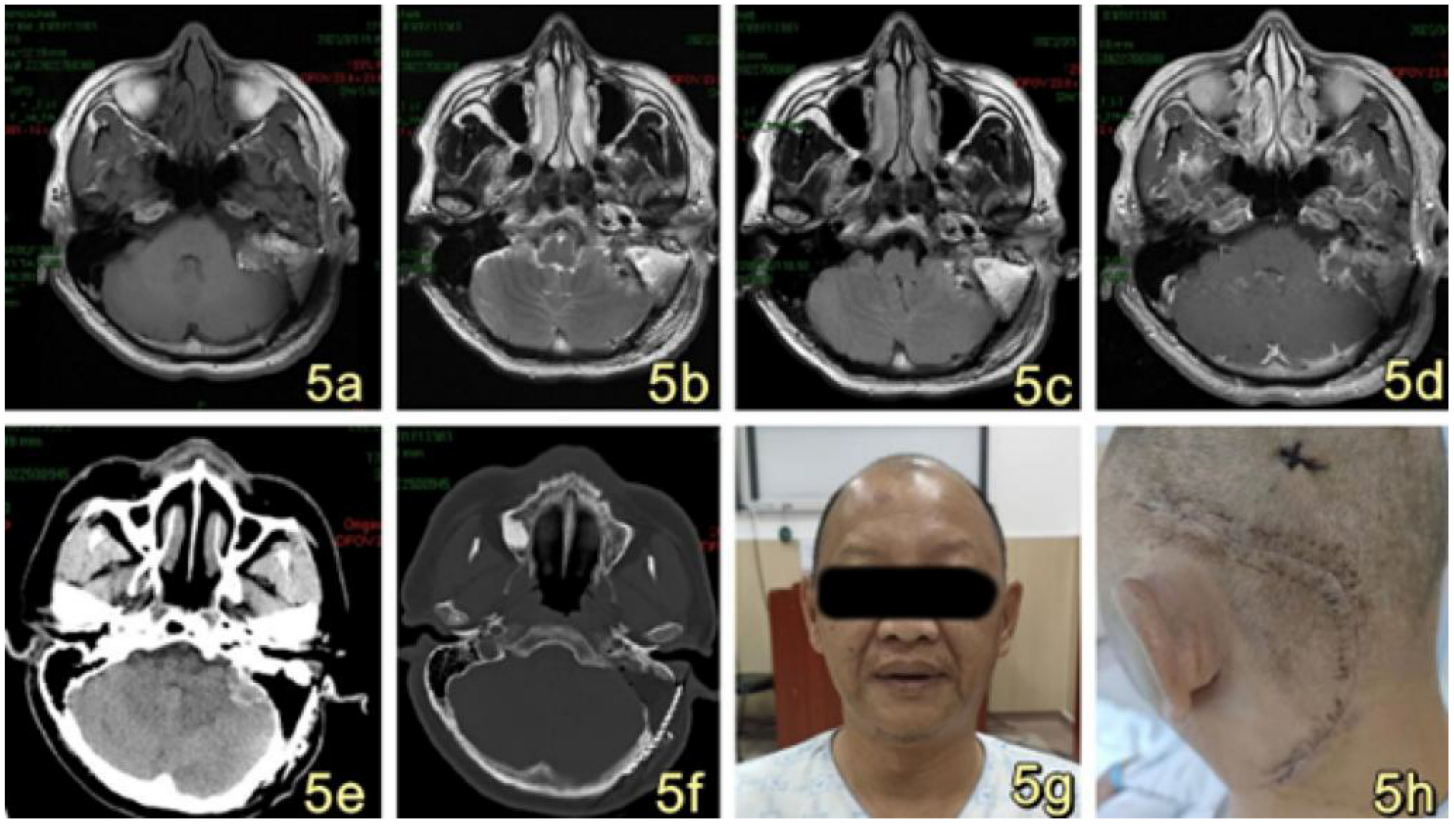
**(A–D)** MRI imaging reveals postoperative alterations in the left pontine cerebellar horn region, characterized by a diminished irregular sigh in comparison to the previous scan. The current extent of the mass measures approximately 26*17mm, accompanied by a thin layer of residual tumor. A new patchy short T1 signal **(A)** and long T2 signal **(B, C)** was observed in the surgical site, along with postoperative changes in the left mastoid and the presence of a fat filling the area. The enhancement scan of the operation area exhibited uneven enhancement, primarily characterized by marginal enhancement **(D)**. **(E, F)** Postoperative CT scan revealed postoperative alterations in the left pontine cerebellar peduncle region, with persisting slightly hyperdense measuring approximately 27*17mm. Additionally, the left mastoid exhibited deformity, accompanied by the presence of fat measuring. **(G)**: The patient exhibited symmetrical frontal striae and nasolabial folds bilaterally following the surgical procedure, with no evidence of asymmetrical mouth angle or facial paralysis. **(H)**: The surgical wounds were closed in a cosmetically pleasing manner and exhibited satisfactory healing along the incision line.

Postoperative evaluation of cranial MRI revealed the presence of a residual thin layer of tumor (**Figures 5A–D**). Additionally, CT imaging indicated the presence of a mixed slightly hyperdense in the left pontine cerebellar peduncle area, measuring approximately 27*17mm in size. Furthermore, a fat density was observed in the left mastoid process (**Figures 5E, F**). Facial auditory nerve and meridian injury were successfully prevented, and adjuvant gamma knife radiotherapy was administered to address the residual tumor layer. In the immediate postoperative phase, the patient did not exhibit any neurological impairment. Furthermore, there was bilateral symmetry of the frontal lines and nasolabial folds, with no evidence of facial asymmetry or paralysis (**Figure 5G**). Additionally, there were no indications of cerebrospinal fluid leakage, and the wound along the incision site showed satisfactory healing (**Figure 5H**). The patient was discharged from the hospital 17 days following the surgical procedure. On the 40th day post-operation, a whole-body PET-CT tomography scan was conducted. The results indicated postoperative alterations in the left pontine cerebellar horn region. Specifically, changes were observed in the left mastoid process, with the presence of fat filling the left mastoid process. Additionally, the left pontine cerebellar horn pool still contained mixed slightly high-density. Notably, the size of the left pontine cerebellar horn pool had significantly reduced to approximately 9×7mm compared to its preoperative size. No anomalous density was observed in the remaining brain parenchyma. There was no observed expansion or constriction of the ventricles and cerebral pools. The sulcus and fissure exhibited distinct clarity, with no observed signs of abnormal widening. The midline structure of the brain exhibited central alignment. The evaluation of the MRI scan conducted during the interval revealed that the mass exhibited no signs of progression and did not give rise to any complications. The procedure did not cause any medical nerve damage.

**Figure 6:**
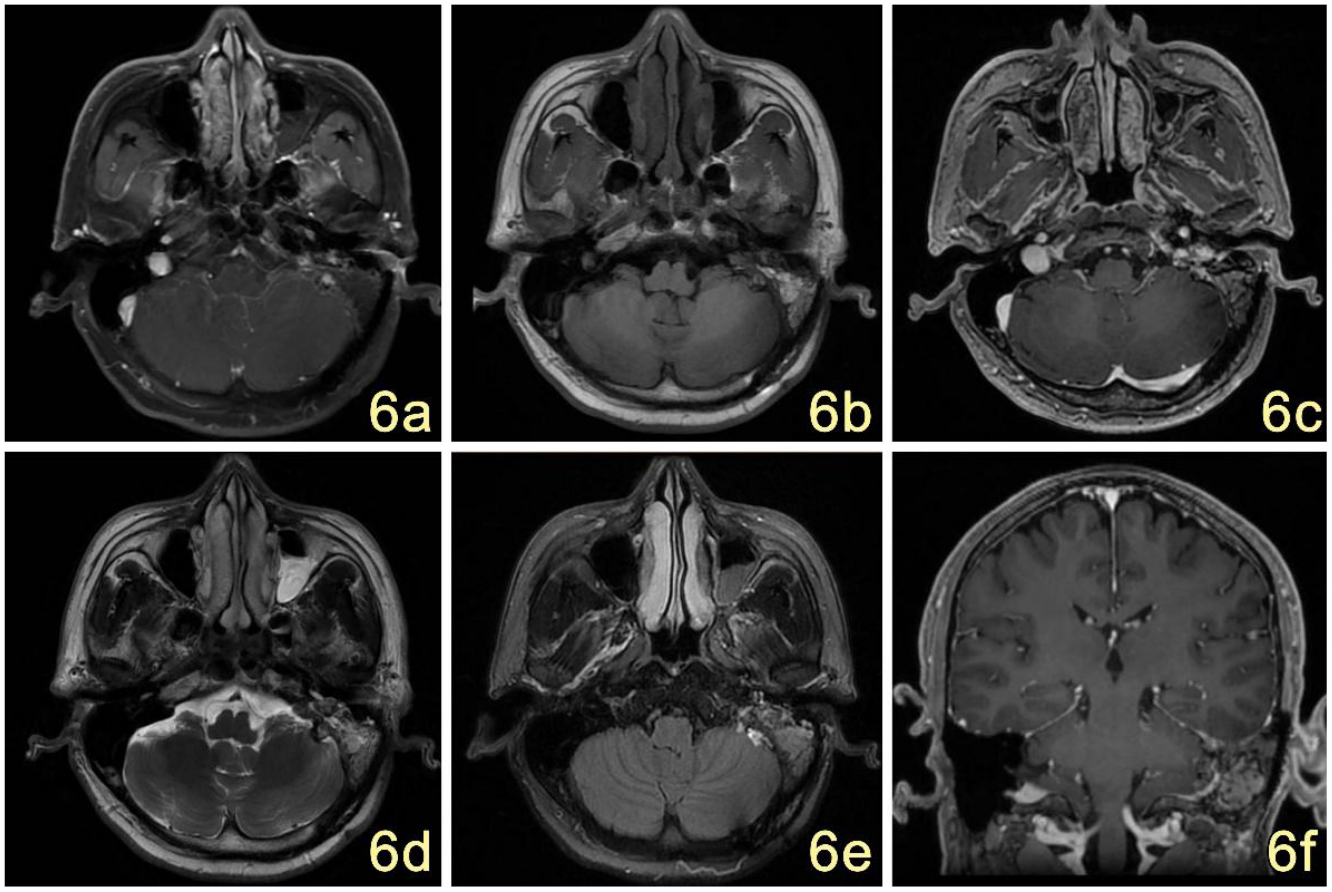
**(A–F)** MRI showed postoperative changes in the mastoid process of the left temporal bone, with irregular mixed-signal in the T1W1 **(B)**, T2W1 **(D)**, and FLAIR **(E)** areas, which were significantly reduced in extent compared with the previous ones, and the edges of the surgical area were little enhanced on the enhancement scans **(C)**; no abnormal signal were seen in the adjacent soft tissues. The signal of the left mastoid process was increased and narrowed **(F)**.

**Figure 7:**
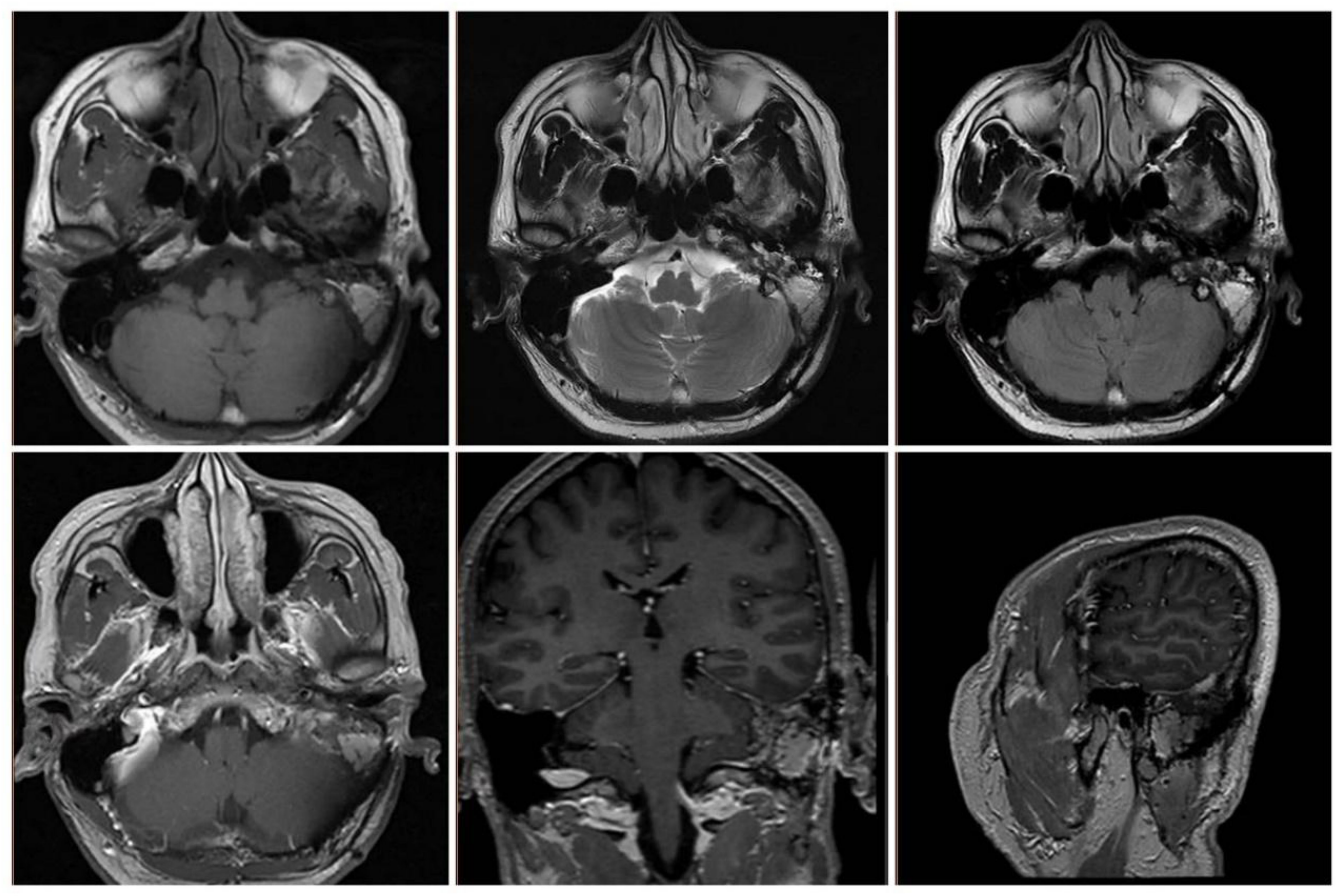
MRI at 15 months postoperatively showed stabilization of the residual lesion and patency of the jugular vein.

Postoperatively, the patient underwent gamma knife-assisted therapy. The latest MRI examination, conducted 9 months after surgery, revealed a significant reduction in the tumor mass. No abnormal signal foci were detected in the adjacent soft tissues of the mastoid process of the left temporal bone. Residual tumor at the nodal level in the left pontine cerebellar horn region, primarily presenting as a mixed lesion with a patent jugular venous area and irregular enhancement of the margins on contrast-enhanced scans. (**Figures 6A–F**). As of the last follow-up, conducted 18 months after the surgical intervention, the patient exhibited no signs of facial paralysis or any vestibular symptoms indicative of tumor recurrence. VHL gene sequencing and the associated diagnostic evaluations were not performed due to the patient’s socioeconomic status. An MRI conducted 15 months post-surgery revealed no progression of the tumor, as illustrated in **Figure 7**.

## Discussion

Endolymphatic sac tumor (ELST) is an uncommon epithelial neoplasm originating from the endolymphatic ducts and endolymphatic sacs. It was initially characterized as an adenomatous tumor of the endolymphatic sac by Hassard et al. (4) in 1984. The interstitium of this structure exhibits a high density of blood vessels and is distinguished by its papillary and glandular tubular morphology. Although the disease is histologically benign, it has the ability to infiltrate and destroy bone locally. Clinically, it exhibits an aggressive behavior, although no cases of metastasis have been reported. This characteristic sets it apart from other adenomatous tumors of the middle ear epithelium (5). The main clinical presentation is characterized by progressive sensorineural hearing loss, with or without cranial neuropathy. This is believed to be caused by tumor progression, which exerts pressure on adjacent auditory structures or leads to endolymphatic edema (6). The severity of symptoms also depends on the location and extent of the affected brain tissue. Tumors typically infiltrate neighboring structures situated in four orientations: lateral, medial, superior, and anterior. The tumor progresses towards the middle ear through the mastoid pathway, causing erosion of the vestibule, posterior semicircular canals, and mastoid cavity (7). Additionally, it may also affect the jugular bulb and facial nerve (8). Tumor infiltration into the pontocerebellar horn region or the posterior cranial fossa is a frequently observed pattern of tumor growth (9).

ELST exhibits a broad range of age of onset, spanning from 15 to 77 years, with the majority of diagnoses occurring in individuals in their 50s or 60s (10). There are no significant gender differences in the occurrence of ELST. VHL is attributed to germline mutations in the tumor suppressor gene located on chromosome 3P25-26. Genetic pathogenesis and molecular studies of VHL disease-associated endolymphatic sac tumors (ELST) have revealed that their mutations are akin to those found in other tumors linked to VHL disease (11). Consequently, ELST has been recognized as one of the pathologies associated with VHL disease. The incidence of endolymphatic sac tumors in patients with VHL ranges from 2% to 11% (12). Additionally, there is a 5% to 20% likelihood of these tumors progressing to ELST. ELST is predominantly bilateral, with a higher incidence in females, and tends to be diagnosed at an earlier age (13). Preoperative testing for VHL genetic mutations, as well as fundus and abdominal ultrasonography or positron emission computed tomography (PET-CT), should be conducted as a standard practice for all patients diagnosed with or suspected of having endolymphatic sac tumor (ESLT). This is necessary to rule out the presence of VHL syndrome in other areas of the body. Our patient’s perfect PET-CT did not reveal any associated tumor. We advised the patient to test for the VHL gene and the patient may be unable to cooperate due to personal circumstances.

The degree of functional impairment associated with tumor extension is contingent upon the location affected by the tumor. Patients frequently exhibit sensorineural hearing loss (94%), ataxia (62%), ear swelling, tinnitus (55%), vertigo (47%), and facial palsy (33%) (10, 14, 15), which closely resemble the symptoms of Ménière’s disease. Moreover, larger tumors with a greater diameter may manifest additional cranial neurologic lesions, including dysphagia, choking, hoarseness, and diplopia. Physical examination findings indicate an intact tympanic membrane, while the presence of an erythematous mass located posterior to the tympanic membrane suggests the involvement of the middle ear in External Auditory Canal Lymphoma (ELST). It is important to distinguish this condition from other potential differential diagnoses such as tympanic ventricular body tumors and jugular vein globe tumors (16). The latter exhibits a more abundant blood supply, often characterized by a cherry red coloration, and is commonly linked to the presence of pulsatile tinnitus. When the tumor affects the facial nerve and the region of the jugular foramen, it is important to carefully evaluate the functionality of the facial nerve and the posterior group of cranial nerves.

The preoperative evaluation and differential diagnosis of Endolymphatic Sac Tumors (ELST) relies on radiology. Computed Tomography (CT) imaging enables the visualization of characteristic bone destruction associated with ELST. The tumor is typically situated in the region between the posterior medial petrous bone and the sigmoid sinus. The presence of an irregular thin calcified rim at the posterior margin of the tumor plays a crucial role in the diagnostic process. The involvement of the posterior wall of the internal auditory canal, jugular bulbous fossa, pontine cerebellar angle pool, middle ear, cavernous sinus, and pterygoid sinus can occur when the volume is large. Magnetic Resonance Imaging (MRI) reveals that the hemorrhagic area, containing methemoglobin and ferritin-containing precipitates, exhibits a significant high signal on T1-weighted imaging (T1WI) and T2-weighted imaging (T2WI). Low signal areas exhibit a response to localized necrosis, calcification, or residual bone. Tumors larger than 2 cm in size may exhibit blood flow cavities. Digital subtraction angiography (DSA) helps to rule out intracranial vascular invasion (1), and the possibility of preoperative embolization can be explored in this highly vascularized tumor, which is essential for safe surgical planning. In our patient, preoperative refinement of the DSA examination suggested a blood supply from the left ascending pharyngeal artery, and the tumor staining was evident, indicating that the tumor had a rich blood supply, as observed in the single-branch supply. This information also played a crucial role in shaping the design and approach of the surgical plan in this case. One distinguishing characteristic of endolymphatic sac tumors (ELST) is that their main blood supply arteries originate from the external carotid artery system, including the ascending pharyngeal artery, superficial temporal artery, occipital arteries, and middle meningeal artery (17). The anterior inferior cerebellar arteries may also be involved in the blood supply. ELST can be challenging to diagnose as it shares similar features with intrinsic temporal bone tumors and post-labyrinthine lesions, such as paragangliomas, choroid plexus papillomas, and distant metastatic disease (18). Despite the presence of clinical and imaging features, distinguishing ELST from other lesions remains difficult, and histopathological examination is necessary for a definitive diagnosis.

Pathohistologically, epithelioid hemangioendotheliomas (ELSTs) are predominantly characterized by red or dark purple polypoid lesions. These lesions are typically soft in texture and may occasionally exhibit the presence of bone fragments, lacking an intact peritoneum. Tumors are typically papillary and cystic adenoid (19), and the tissue sent from this patient exhibited papillary and cystic adenoid structures microscopically. The surface is covered with a single layer of cuboidal or flat epithelium, and the mesenchymal areas are rich in blood vessels and may be filled with colloid cyst-like structures (6). Cytologic observations reveal infrequent occurrences of mitoses, with no evidence of necrotic regions. In the field of immunohistochemistry, the epithelial membrane antigen (EMA), CK5/6+, and neurospecific enolase (NSE) exhibited positive staining.

The International Union Against Cancer (UICC) has not yet implemented a clinical grading system for Endolymphatic Sac Tumors (ELST). In 2004 and 2006, Bambakidis (20) and Schipper (21) proposed a clinical grading system for ELST. However, this grading system was unable to distinguish between patients who had the potential to preserve residual hearing. Additionally, the staging description was not adequately detailed and did not encompass tumors with intracranial invasion. As a result, the proposed grading system did not provide comprehensive guidance to surgeons in selecting the appropriate surgical approach. The aforementioned limitation hinders its ability to provide adequate guidance to surgeons in selecting the most suitable surgical approach. Therefore, Li.F et al. (22) proposed a novel grading system for tumors located in the petrous bone. Grade I tumors are confined to the posterior margin of the petrous bone, between the posterior semicircular canals and the posterior cranial fossa. These tumors may also infringe on the facial nerve, but do not involve the inner ear and jugular foramen. Grade II tumors extend forward to involve the semicircular canals, cochlea, inner auditory canal, or middle ear, but do not affect the pontine cerebellar angle and jugular foramen. Grade IIIa tumors invade the jugular foramen, internal carotid artery, or the ramus. Grade IIIb tumors infiltrate the intracranium, cavernous sinus, and/or jugular foramen. The grading system described in this study encompasses the evaluation of External Lateral Skull Tumors (ELSTs) at various stages of growth, ranging from initial confinement to the posterior border of the temporal bone rock to potential intracranial invasion.

Microsurgery continues to serve as the fundamental approach for treatment, with the surgical objective being the complete excision of the tumor while preserving the patient’s auditory and facial nerve function to the greatest extent possible. Although still a subject of debate, certain tumors may necessitate the administration of preoperative or postoperative radiotherapy (23). Additionally, highly vascularized tumors may be subjected to preoperative embolization (24). In this patient, intraoperative bleeding from the tumor was evident, and the surgical team reflected on the potential to reduce intraoperative bleeding as well as the difficulty of the procedure if effective preoperative embolization of the supplying artery had been performed. The selection of the operative modality should be based on the preoperative assessment of hearing and the clinical grading of the tumor. Grade I refers to a tumor that is localized in the posterior region of the labyrinth. Typically, patients with Grade I tumors experience mild to moderate hearing loss. To preserve any remaining hearing in this patient group, the recommended treatment is posterior labyrinthine pathway + posterior rock bone resection. Grade II tumors primarily invade the anterior region but remain localized within the temporal bone. At this stage, the tumor involves the labyrinth or internal auditory canal, resulting in significant hearing loss for the patient. To achieve complete tumor removal, the transvaginal pathway temporal bone subtotal resection is typically selected. In the third grade, the tumor infiltrates the structures outside the temporal region. Grade IIIa specifically affects the structures associated with the jugular foramen, and it is recommended to use the infratemporal fossa approach for tumor resection. Grade IIIb, on the other hand, involves the intracranial area. In such cases, the infratemporal fossa approach is typically combined with either the posterior ethmoidal sinus approach or the middle cranial fossa approach. If the tumor extends to the jugular vein bulb and there is a high risk of bleeding, intraoperative bleeding from the subxiphoid sinus can be managed by employing the method of filling the subxiphoid sinus with the sigmoid sinus tunnel after occluding the sigmoid sinus and ligating the internal jugular vein. This technique allows for the avoidance of injury to the posterior group of cerebral nerves while resecting the lateral wall of the jugular vein bulb and the tumor. If the tumor extends into the external auditory canal and the diameter of the intracranial tumor exceeds 2 cm, it is recommended to perform a second-stage resection of the intracranial tumor to prevent cerebrospinal fluid leakage. In this case, the tumor measured approximately 28x21x27mm, extending towards the pontine cerebellar angle area and encroaching upon the jugular foramen area. Due to the location and size of the tumor, the surgical team decided to use a combined distal lateral and anterior ethmoidal sinus approach to achieve a total resection of the tumor in both the intracranial and extracranial portions of the skull.

No instances of recurrence have been documented subsequent to the successful surgical removal of ELST. However, it is important to note that partial resection may result in local recurrence. Additionally, achieving total resection of advanced ELST is often unattainable due to the intricate anatomical nature of the tumor and its diverse expansion patterns. Revised 2: Recurrence of eosinophilic granuloma-like lesion of the skull (ELST) is more frequent compared to other major osteolytic bone tumors. One common cause of recurrence is the improper resection of the affected dura mater (25). Gioacchini et al. (26) conducted a study with a mean follow-up period of 49.7 months (ranging from 8 to 136 months) and reported a pooled proportion of tumor recurrence (95% confidence interval) of 14.0% (9.7-19.3). Tang et al. (13) analyzed 253 discrete tumors and found recurrence in 16.2% of cases, with 10.6% of cases showing progression of residual lesions after treatment. The average duration until recurrence or progression was 53.1 ± 52.4 months, with a range of 3 to 240 months. There is empirical evidence suggesting that local radiation therapy yields superior clinical outcomes for residual and recurrent lesions measuring less than 3 cm, particularly when employing gamma-ray stereotactic radiosurgery (27).

## Conclusion

We hereby present this exceptional case in order to enhance our comprehension and recognition of this exceedingly uncommon neoplasm. Endolymphatic sac tumors require a high degree of vigilance for early diagnosis, and complete resection of the lesion is preferred while maintaining a normal neurological status. Proper and rational preoperative planning, such as embolization in specific cases, will facilitate surgery, avoid intraoperative bleeding. After confirming the histopathological diagnosis, radiotherapy and Gamma Knife radiation therapy may be options for treating residual tumors (28), which play an important role in controlling tumor recurrence. Given the potential presence of residual mass and the risk of recurrent disease, it is crucial to conduct long-term follow-up. This should involve regular magnetic resonance imaging (MRI) every 6 months during the initial period, followed by annual MRI scans for a duration of 10 years (29).

## Data Availability

The original contributions presented in the study are included in the article/supplementary material. Further inquiries can be directed to the corresponding author/s.
